# Profiling of Oral Microbiota and Cytokines in COVID-19 Patients

**DOI:** 10.3389/fmicb.2021.671813

**Published:** 2021-07-30

**Authors:** Valerio Iebba, Nunzia Zanotta, Giuseppina Campisciano, Verena Zerbato, Stefano Di Bella, Carolina Cason, Roberto Luzzati, Marco Confalonieri, Anna Teresa Palamara, Manola Comar

**Affiliations:** ^1^Department of Medical, Surgical, and Health Sciences, University of Trieste, Trieste, Italy; ^2^Laboratory of Advanced Microbiology Diagnosis and Translational Research, Institute for Maternal and Child Health IRCCS Burlo Garofolo, Trieste, Italy; ^3^Infectious Diseases Department, University of Udine, Udine, Italy; ^4^Pulmonology Department, University Hospital of Cattinara, Trieste, Italy; ^5^IRCCS San Raffaele Pisana, Rome, Italy; ^6^Laboratory Affiliated to Institute Pasteur Italia- Cenci Bolognetti Foundation, Department of Public Health and Infectious Diseases, Sapienza University of Rome, Rome, Italy

**Keywords:** microbiota (D064307), cytokines, machine learning, oral microbiota, network analysis, COVID-19, metagenomics

## Abstract

The presence of severe acute respiratory syndrome coronavirus 2 (SARS-CoV-2) has been recently demonstrated in the sputum or saliva, suggesting how the shedding of viral RNA outlasts the end of symptoms. Recent data from transcriptome analysis show that the oral cavity mucosa harbors high levels of angiotensin-converting enzyme 2 (ACE2) and transmembrane protease, serine 2 (TMPRSS2), highlighting its role as a double-edged sword for SARS-CoV-2 body entrance or interpersonal transmission. Here, we studied the oral microbiota structure and inflammatory profile of 26 naive severe coronavirus disease 2019 (COVID-19) patients and 15 controls by 16S rRNA V2 automated targeted sequencing and magnetic bead-based multiplex immunoassays, respectively. A significant diminution in species richness was observed in COVID-19 patients, along with a marked difference in beta-diversity. Species such as *Prevotella salivae* and *Veillonella infantium* were distinctive for COVID-19 patients, while *Neisseria perflava* and *Rothia mucilaginosa* were predominant in controls. Interestingly, these two groups of oral species oppositely clustered within the bacterial network, defining two distinct Species Interacting Groups (SIGs). COVID-19-related pro-inflammatory cytokines were found in both oral and serum samples, along with a specific bacterial consortium able to counteract them. We introduced a new parameter, named CytoCOV, able to predict COVID-19 susceptibility for an unknown subject at 71% of power with an Area Under Curve (AUC) equal to 0.995. This pilot study evidenced a distinctive oral microbiota composition in COVID-19 subjects, with a definite structural network in relation to secreted cytokines. Our results would be usable in clinics against COVID-19, using bacterial consortia as biomarkers or to reduce local inflammation.

## Introduction

Coronavirus disease 2019 (COVID-19) is a global pandemic established at the end of 2019, whose etiological agent is severe acute respiratory syndrome coronavirus 2 (SARS-CoV-2), a member of betacoronaviruses (Li et al., 2020). COVID-19 in the more severe disease is typically characterized by: (i) symptoms of the lower respiratory tract (Srivastava and Gupta, 2020); (ii) a systemic “cytokine storm” (de la Rica et al., 2020; Jose and Manuel, 2020); and (iii) ageusia and hyposmia (Gupta et al., 2020; Srivastava and Gupta, 2020). Patients exhibiting an exaggerated form of symptoms (Wang et al., 2020) showed greater levels of pro-inflammatory factors (Chen et al., 2020a; Li et al., 2020; Qin et al., 2020), and besides respiratory illnesses, they may also have enteric symptoms and encephalitis (Gupta et al., 2020). Virus replication in the throat particularly during the first 5 days of symptoms is strongly supported by identification of transcribed subgenomic mRNA in throat swab samples. However, some reports suggest the potential for pre- or oligosymptomatic transmission as a consequence of a mild illness of the upper respiratory tract. The presence of SARS-CoV-2 has been recently demonstrated in the sputum or “posterior oropharyngeal saliva” (Braz-Silva et al., 2020; Leung et al., 2020; To et al., 2020a, b), indicating that shedding of viral RNA outlasted the end of symptoms. Transcriptome analysis found that angiotensin-converting enzyme 2 (ACE2) and transmembrane protease, serine 2 (TMPRSS2) receptors, employed by SARS-CoV-2 to enter cells, were highly expressed in salivary glands and epithelial cells, showing the potential vulnerability risk for oral cavity for lung or gut involvement (Herrera et al., 2020). From these data, virus–host interplay within the oral cavity seems to be a promising feature of COVID-19 pathogenesis, forming the basis of disease severity and spread. The relationship between virus and host environment included disturbance of resident bacterial community, as recently reported within the gut of COVID-19 patients (Feng et al., 2020). It is also true that oral, lung and gut microbiota are intermingled in their functions through yet unknown mechanisms, and that oral dysbiosis (triggered or maintained by SARS-CoV-2 infection) could be spread to the other two body districts (Bao et al., 2020; Xiang et al., 2020). In this proposition, it is known that oral pathogens involved in periodontal diseases or present on the tongue dorsum (e.g., *Prevotella* and *Veillonella* genera) take advantage of local dysbiosis and, when dispersed through the blood stream or inhaled, could propagate and instaurate a new disease in distant organs, such as heart and lung (Leishman et al., 2010; Mammen et al., 2020). In this view, the oral cavity could act as a potential reservoir not only for the SARS-CoV-2 but also for a dysbiotic microbiota with a lung pathogenic potential (Bao et al., 2020; Xiang et al., 2020), Thus, characterizing the oral microbiota structure when SARS-CoV-2 is present may identify physiological markers for the potential risk in terms of disease severity and therapeutic strategies. In the present study, we characterized the interplay of oral microbiota and inflammatory cytokines in COVID-19 patients.

## Materials and Methods

### Study Cohort and Samples

A total of 26 patients, 6 women (mean age 66 ± 16 years) and 20 men (mean age 66 ± 15 years) hospitalized at the Infectious Diseases Unit, University of Trieste, Italy, between April 10, 2020 and May 5, 2020, tested positive for COVID-19, were selected for this study. All patients provided informed consent for the use of their data and clinical samples for the purposes of the present study. All patients did not take antibiotics or probiotics in a 3-month period before sampling. Patients acquired their infections upon known close contact to an index case, thereby avoiding representational biases owing to symptom-based case definitions. All patients had interstitial pneumonia and were receiving oxygen therapy but did not require endotracheal intubation and invasive mechanical ventilation (Raghu and Wilson, 2020; Wong et al., 2020). Oropharyngeal and nasopharyngeal swabs for diagnosis of SARS-CoV-2 were performed, and oral swab specimens touching the tongue, palatum and cheeks were additionally collected for oral microbiota and local immune response profiling. All samples were taken before starting any therapy against COVID-19. No mouth washing products were administered to the patients. Specimens were additionally collected with the same modality from age-matched healthy volunteers (*n* = 15) without evaluable risk for SARS-CoV-2 infection and without antibiotics or probiotics intake in a 3-month window before sampling. In addition, sera samples from 11 infected patients with severe disease who underwent endotracheal intubation and invasive mechanical ventilation were analyzed for peripheral cytokines profile.

### DNA and RNA Extraction

Total DNA and RNA were extracted starting from 300 and 200 μl of samples, respectively, in a final elution volume of 50 μl, using the Maxwell CSC Instrument (Promega Srl, Italy) and following the manufacturer’s instructions.

### SARS-CoV-2 Detection

SARS-CoV-2 detection was performed on the CFX96^TM^ Real-Time PCR Detection System (Bio-Rad, Hercules, CA, United States), using the NeoPlex^TM^ COVID-19 Detection Kit (GeneMatrix, Seongnam, Kyonggi-do, South Korea) targeting viral *N* and *RdRp* genes and the housekeeping gene of β-actin as internal control, following the manufacturer’s instructions.

### ACE2 and TMPRSS2 Expression

The expression levels of human *ACE2* and *TMPRSS2* genes were evaluated by SYBR green PCR analyses. In brief, RNA was reverse transcribed using the SensiFast cDNA Synthesis Kit (Bioline, Taunton, MA, United States), and SYBR green PCR analysis was performed using the Kapa HiFi HotStart Ready Mix (Roche Diagnostics Deutschland, Mannheim, Germany) (Ma et al., 2020). The housekeeping Beta-globin human gene was used for normalization, and the relative expression levels (ΔCt) of human *ACE2* and *TMPRSS2* genes were compared between groups.

### 16S-Targeted Sequencing

The V2–V3 portion of the 16S rRNA was amplified, using the primer set F101-R534, with a different IonXpress barcode per sample attached to the reverse primer. PCR reactions were performed using the Kapa Library Amplification Kit (Kapa Biosystems, Wilmington, MA, United States) and BSA 400 ng/μl, under the following conditions: 5 min at 95°C, 30 s at 95°C, 30 s at 59°C, 45 s at 72°C, and a final elongation step at 72°C for 10 min. DNA after normalization was quantified with a Qubit^®^ 2.0 Fluorometer (Invitrogen, Carlsbad, CA, United States). The amplicon size was checked on a 2% agarose gel. The subsequent step of PCR purification was carried out using the Mag-Bind^®^ Total Pure NGS beads (OMEGA Bio-Tek, Norcoss, GA, United States), retaining fragments > 100 bp. Template preparation was performed by the Ion PGM Hi-Q View kit on the Ion OneTouch^TM^ 2 System (Life Technologies, Grand Island, NY, United States) and sequenced using the Ion PGM Hi-Q View sequencing kit (Life Technologies, Grand Island, NY, United States) with the Ion PGM^TM^ System technology. Negative controls, including a no-template control, were processed with the clinical samples (Campisciano et al., 2017).

### Microbiota Characterization

Raw FASTQ files were analyzed with DADA2 pipeline v.1.14 for quality check and filtering (sequencing errors, denoising, chimera detection) on a Workstation Fujitsu Celsius R940 (Fujitsu, Tokyo, Japan) (Supplementary Figure 1). Filtering parameters were as follows: truncLen = 0, minLen = 100, maxN = 0, maxEE = 2, truncQ = 11 and trimLeft = 15. All the other parameters in the DADA2 pipeline for single-end IonTorrent were left as default. Raw reads (2,447,325 in total, on average 59,691 per sample) were filtered (818,531 in total, on average 19,964 per sample), and 962 Amplicon Sequence Variants (ASVs) were found. Sample coverage was computed and resulted to be on average higher than 99% for all samples, thus meaning a suitable normalization procedure for subsequent analyses. Bioinformatic and statistical analyses on recognized ASVs were performed with Python v.3.8.2. Each ASV sequence underwent a nucleotide Blast using the National Center for Biotechnology Information (NCBI) Blast software (ncbi-blast-2.3.0) and the latest NCBI 16S Microbial Database accessed at the end of July 2020 (ftp://ftp.ncbi.nlm.nih.gov/blast/db/). After blasting, the 962 ASVs were merged into 122 species (thus excluding sub-species or strain differences), and a matrix of their relative abundances was built for subsequent statistical analyses.

### Network Analysis

Pearson matrices for network analysis (metric = Bray–Curtis, method = complete linkage) were generated on normalized and standardized data with in-house scripts (Python v3.8.2) and visualized with Gephi v.0.9.2, as previously reported (Derosa et al., 2020; Riganelli et al., 2020). Bacterial species having a prevalence ≥ 5% were considered to generate the nodes within the final network, while a significant Pearson correlation coefficient and its related *p*-value (after Benjamini–Hochberg FDR at 10%) was employed to obtain eight categories defining edge thickness (Li et al., 2008). A leave-one-out method was employed by SciKit-learn package v0.4.1 on the subjects in order to have an averaged *p*-value for each correlation among two definite variables. Network analysis was performed on unified datasets (Derosa et al., 2020) taking care of an optimized visual representation with Gephi v.0.9.2, as proposed by current guidelines (Merico et al., 2009; Faust and Raes, 2012; Faust et al., 2012; Lozupone et al., 2012; Berry and Widder, 2014). Nodes were colored according to the cohort in which species harbored the highest mean relative abundance, after normalization and standardization. The degree value, measuring the in/out number of edges linked to a node, and the betweenness centrality, measuring how often a node appears on the shortest paths between pairs of nodes in a network, were computed with Gephi v.0.9.2. Intranetwork communities (here called Species Interacting Groups—“SIGs” (Iebba et al., 2018; Derosa et al., 2020) were retrieved using the Blondel community detection algorithm (Blondel et al., 2008) by means of randomized composition and edge weights, with a resolution equal to 1 (Lambiotte et al., 2014).

### Soluble Immune Mediators Quantification

The profile of a panel of 27 cytokines including chemokines and growth factors was assessed in duplicate, in oral swabs of positive and negative subjects for SARS-CoV-2 using magnetic bead-based multiplex immunoassays (Bio-Plex Pro^TM^ human cytokine 27-plex panel; Bio-Rad Laboratories, Milan, Italy) according to the pre-optimized protocol (Zanotta et al., 2015). Briefly, the undiluted samples (50 μl) were mixed with biomagnetic beads in 96-well flat-bottom plates, and after incubation for 30 min at room temperature followed by washing the plate with Bio-Plex wash buffer, 25 μl of the antibody–biotin reporter was added. After the addition of 50 μl of streptavidin–phycoerythrin (PE) and following incubation for 10 min, the concentrations of the cytokines were determined using the Bio-Plex-200 system (Bio-Rad Corp., Hercules, CA, United States) and Bio-Plex Manager software (v.6; Bio-Rad). The data were expressed as median fluorescence intensity (MFI) and concentration (pg/ml).

### Statistical Analysis

Data matrices (microbiota taxa or cytokines) were firstly normalized then standardized using QuantileTransformer and StandardScaler methods from Sci-Kit learn package v0.20.3. Normalization using the output_distribution = “normal” option transforms each variable to a strictly Gaussian-shaped distribution, while the standardization results in each normalized variable having a mean of zero and variance of one. These two steps of normalization followed by standardization ensure the proper comparison of variables with different dynamic ranges, such as bacterial relative abundances or cytokines levels. For microbiota analysis, measurements of α diversity (within sample diversity), such as Richness and Shannon index, were calculated at species level using the SciKit-learn package v.0.4.1. Exploratory analysis of β-diversity (between sample diversity) was calculated using the Bray–Curtis measure of dissimilarity and represented in Principal Coordinate Analysis (PCoA), along with methods to compare groups of multivariate sample units (analysis of similarities—ANOSIM, permutational multivariate analysis of variance—PERMANOVA) to assess significance in data points clustering (Anderson and Walsh, 2013). ANOSIM and PERMANOVA were automatically calculated after 999 permutations, as implemented in SciKit-learn package v0.4.1. We implemented Partial Least Square Discriminant Analysis (PLS-DA) and the subsequent Variable Importance Plot (VIP) as a supervised analysis wherein the VIP values (order of magnitude) are used to identify the most discriminant bacterial species among COVID-19 and control samples. Bar thickness reports the fold ratio (FR) value of the mean relative abundances for each species among the two cohorts, while an absent border indicates mean relative abundance of zero in the compared cohort. In order to compare the microbiota species with cytokines levels, a multivariate statistical Pearson correlation analysis (and related *p*-values) was performed with custom scripts (Python v3.8.2), and a Hierarchical Clustering Analysis (HCA) with “Bray–Curtis” metrics and “complete linkage” method was used to visualize putative cross-correlation clusters. Mann–Whitney U and Kruskal–Wallis tests were employed to assess significance for pairwise or multiple comparisons, respectively, considering a *p*-value < 0.05 as significant. Statistical analyses gathering more than two groups were performed using ANOVA followed by pairwise comparisons with Bonferroni adjustments. Differential enrichment analyses in murine studies were corrected for multiple hypothesis testing using a two-stage Benjamini–Hochberg FDR at 10%.

### Data Availability

Raw fastq files were submitted to NCBI Sequence Read Archive (SRA) portal under the Bioproject PRJNA692359, submission SUB8898095.

## Results

Data relative to demographics and clinical data of enrolled patients at the time of samples collection are resumed in Table 1. Cardiac dysfunctions (14/26) and neurological involvement (11/26) including ageusia or hyposmia (9/26) and paralysis and epilepsy (2/26) represent the most frequently present comorbidities. Regarding drug therapies, all patients were treated, after sampling, with hydroxychloroquine, and 57.7% of them (15/26) received combinations with antibiotics.

**TABLE 1:** Patients’ demographics.

**Patients**	**Men (*n* = 21)**	**Women (*n* = 5)**	**Total (*n* = 26)**
Mean age	66 ± 15.8	72.6 ± 12.9	67.3 ± 15.3

**Symptoms**			

Hyposmia	8/21 (38%)	1/5 (20%)	9/26 (34.6%)
Loss of taste	8/21 (38%)	1/5 (20%)	9/26 (34.6%)
Neurological alterations	1/21 (4.7%)	0	1/26 (3.84%)
Pneumonia	21/21 (100%)	5/5 (100%)	26/26 (100%)

**Comorbidity**			

Diabetes	5/21 (23.8%)	0	5/26 (19.2%)
Hypertension	12/21 (57.1%)	2/5 (40%)	14/26 (53.8%)
Cancer	3/21 (14.2%)	2/5 (40%)	5/26 (19.2%)
Cardiopathy	10/21 (47.6%)	1/5 (20%)	11/26 (42.3%)
Obesity	13/21 (61.9%)	1/5 (20%)	14/26 (53.8%)

### COVID-19 Patients Harbor a Distinctive Oral Microbiota

**FIGURE 1:**
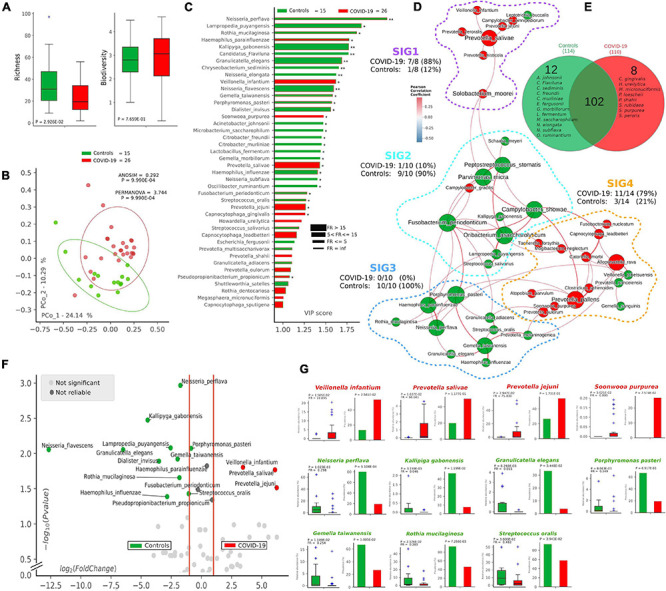
Microbiota composition in COVID-19 and control samples. Alpha- and beta-diversity **(A,B)** of controls (green, *n* = 15) and COVID-19 patients (red, *n* = 26). Variable Importance Plot (VIP, **C**) shows: (i) discriminant species after PLS-DA in descending order of VIP score (bar length); (ii) the highest relative abundance depending on the cohort (central bar color) and the lowest one (edge bar color); (iii) fold ratio (FR) of the highest vs. the lowest relative abundance (bar thickness), and (iv) significant difference after Mann–Whitney *U* test (non-FDR, *p ≤ 0.05, **p ≤ 0.01). Network analysis **(D)** shows communities of bacterial species (namely, species-interacting groups, SIGs) and their positive (red Pearson coefficient) or negative (blue Pearson coefficient) relative abundances correlation. Nodes are colored according to the cohort harboring the higher relative abundance for a definite species, and node name size is directly proportional to the “keystonness” (importance of a species within the overall network). Edge thickness is inversely proportional to the Pearson *p*-value after 10% Benjamini–Hochberg two-stages FDR, and it is colored according to positive (red) or negative (blue) Pearson coefficient. For each SIG are reported percentages of COVID-19- and controls-related species. Venn diagram **(E)** shows species distribution among the two cohorts considering all of the 122 species (not the core microbiota) retrieved by DADA2 pipeline. Volcano plot **(F)** highlights discriminant oral bacterial species in terms of their fold change (*X* axis) and cologarithm of Mann–Whitney *U* test *p*-value (non-FDR) (*Y* axis): species with zero relative abundance were not reported. Pairwise analysis **(G)** of selected 11 species (four for COVID-19—red and seven for controls—green) depicts significant differences in terms of relative abundance and prevalence. In each sub-graph are reported the *p*-value (from Mann–Whitney *U* test) and the fold ratio (FR) among COVID-19 and controls.

Following 16S-targeted sequencing, we observed a significant diminution (-40%) of alpha-diversity (species richness) in COVID-19 patients (*p* = 2.92^∗^10^–2^) (Figure 1A), while Shannon biodiversity was unaltered. The unsupervised algorithm of PCoA visually represented a significant separation of COVID-19 oral samples from controls (*p* < 1^∗^10^–3^) (Figure 1B), thus meaning a different oral microbiota composition assessed with two different measures of beta-diversity (ANOSIM and PERMANOVA). In order to find a pattern of bacterial species able to describe the changes in microbiota composition of COVID-19 samples, we used the supervised algorithm of PLS-DA, which generated a VIP showing the most important species able to separate the two cohorts (Figure 1C). Six bacterial species, having a VIP score higher than the chosen cut-off of 1.25, were discriminant for COVID-19 (*Haemophilus parainfluenzae*, *Veillonella infantium*, *Soonwooa purpurea*, *Prevotella salivae*, *Prevotella jejuni*, and *Capnocytophaga gingivalis*), while several species (*n* = 23) were significantly distinctive for controls (the most important being *Neisseria perflava*, *Lampropedia puyangensis*, *Rothia mucilaginosa*, *Kallipyga gabonensis*, *Candidatus Flaviluna*, and *Granulicatella elegans*). Interestingly, through network analysis, we were able to retrieve four communities, also known as SIGs (Figure 1D), which represent a topological clusterization of bacterial species linked to “functional modules,” for example, a disease status. In order to see if SIGs would be related to COVID-19, nodes were colored according to the cohort in which species had the highest mean relative abundance (after normalization and standardization) (Figure 1D). Two SIGs (SIG1 and SIG4) harbored the majority of COVID-19-related species (18/22, 82%), while SIG2 and SIG3 contained mostly controls species (19/20, 95%), and this repartition of species was significant (Fisher test with Freeman–Halton extension, *p* = 2.13^∗^10^–6^). SIG1 harbored three out of six COVID-19-related species extrapolated from VIP plot (namely, *V. infantium*, *P. salivae*, *P. jejuni*), while *S. purpurea* was included in SIG4, along with other two species known for their noxious effects (*Atopobium parvulum* and *Fusobacterium nucleatum*). The first species depicted by VIP plot as discriminant for COVID-19, namely, *H. parainfluenzae*, was indeed colored as control within the network because of the normalization/standardization procedure, thus meaning that this species would not be reliable as a descriptor. The good community SIG3, harboring three discriminant species for controls (*N. perflava*, *R. mucilaginosa*, and *G. elegans*), and 8 of the overall 23 control-related species, is the farthest from the bad SIG1, probably collecting different genetic and metabolic pathway features with a potential to counteract COVID-19-related species. Among the 122 species retrieved from the DADA2 pipeline (Supplementary Table 1), 102 were shared among the two cohorts, while 12 and 8 were present only in controls and COVID-19 samples, respectively (Figure 1E). Venn diagram relies on the presence of species, not their relative abundance or prevalence; thus, in order to select bacterial species having a plausible and reliable role as biomarkers for COVID-19 and controls, we employed a combination of volcano plot (Figure 1F), species pairwise comparison (Supplementary Figures 2, 3), VIP plot and network analysis, resulting in 11 selected species. Bacterial species biomarkers for COVID-19 are *P. salivae*, *V. infantium*, *P. jejuni* and *S. purpurea* (this latter being present in COVID-19 patients only, Figure 1E). Biomarkers species for healthy oral microbiota are *N. perflava*, *K. gabonensis*, *G. elegans*, *Porphyromonas pasteri*, *Gemella taiwanensis*, *R. mucilaginosa*, and *Streptococcus oralis*.

### Pro-inflammatory Cytokines Are Distinctive for COVID-19 in Both Oral and Serum Samples

**FIGURE 2:**
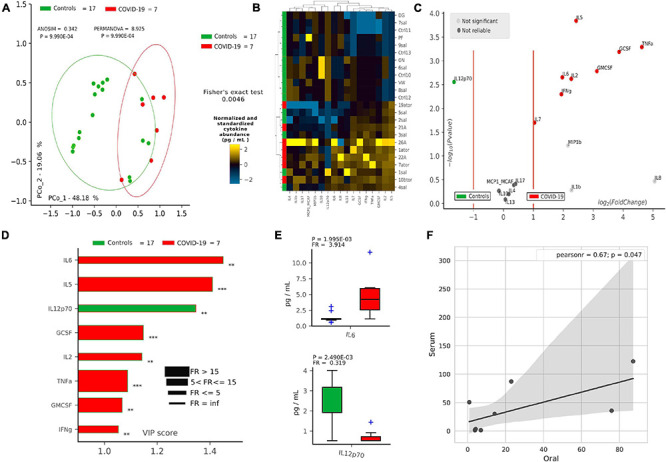
Cytokine pattern in COVID-19 and controls. Cohort separation of controls (green, *n* = 17) and COVID-19 patients (red, *n* = 7) based on cytokine profiles, shown by Principal Coordinate Analysis (PCoA, **A**) and Hierarchical Clustering Analysis (HCA, **B**), following the Bray–Curtis distance algorithm. Volcano plot **(C)** highlights discriminant oral cytokines in terms of their fold change (*X* axis) and cologarithm of Mann–Whitney *U* test *p*-value (non-FDR) (*Y* axis). Variable Importance Plot (VIP, **D**) shows: (i) discriminant cytokines after PLS-DA in descending order of VIP score (bar length); (ii) the highest cytokine quantity (pg/ml) depending on the cohort (central bar color) and the lowest one (edge bar color); (iii) fold ratio (FR) of the highest vs. the lowest cytokine quantity (pg/ml) (bar thickness) and iv) significant difference after Mann–Whitney *U* test (non-FDR, **p ≤ 0.01; ***p ≤ 0.001). Pairwise analysis **(E)** of selected two cytokines depicts significant differences in terms of quantity (pg/ml), reporting *p*-value (from Mann–Whitney *U* test) and fold ratio (FR) among COVID-19 and controls. Pearson linear correlation **(F)** on non-normalized and non-standardized oral (*X* axis) and serum (*Y* axis) cytokines levels (pg/ml), showing significant positive correlation among the two cytokine patterns. Outlier cytokines having extreme values (IL-1Ra, IL-15, and PDGF-bb) were excluded from linear correlation analysis.

After defining oral bacterial species as biomarkers of COVID-19, we investigated their possible correlation with pro-inflammatory cytokines eventually involved in a local “cytokine storm,” as found within patients’ bloodstream recently described in the literature. Using a panel of 27 cytokines including chemokines and growth factors, we found that COVID-19 patients were significantly distinguishable from controls using non-supervised methods, such as PCoA (*p* = 9.9^∗^10^–4^, Figure 2A) and HCA (*p* = 0.0046, Figure 2B). Aiming at finding discriminant oral cytokines for COVID-19 status, we employed volcano plot (Figure 2C) and VIP plot (Figure 2D), finding out seven COVID-19-related discriminant cytokines (IL-6, IL-5, GCSF, IL-2, TNF-α, GMCSF, and INF-γ), while only one (IL-12p70) for controls. IL-6 and IL-12p70 were the most discriminant cytokines for COVID-19 and controls, respectively, as confirmed by their pairwise analysis (Figure 2E and Supplementary Figure 4). Results from serum cytokines profiling from patients with severe symptomatology and complication highlighted a superimposable cytokine profile to the oral one of patients at the onset of infection, resulting in a significant Pearson positive correlation (Figure 2F). In particular, high levels of oral cytokines involved in early antiviral response mirrored cytokine levels in systemic circulation. As reported in the literature for the lung, the expression of human ACE2 and TMPRSS2 in mucosal oral samples was downregulated in infected patients (greater than 100-fold) compared with no infected subjects, and no significant association was found with microbiome composition or cytokines profile (data not shown).

### Oral Bacterial Species Topologically Counteracts COVID-19-Related Cytokines

**FIGURE 3:**
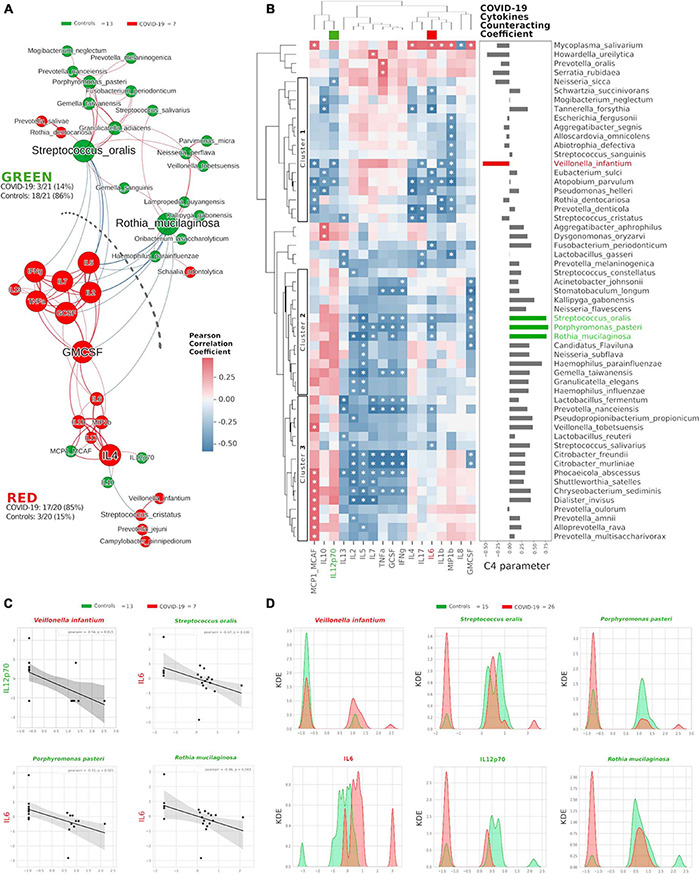
Integration of oral species and cytokines datasets. Network analysis **(A)** shows communities (namely, GREEN for controls and RED for COVID-19) of bacterial species and cytokines and their positive (red Pearson coefficient) or negative (blue Pearson coefficient) abundances (relative percentage or pg/ml, respectively, for species and cytokines) correlation. Nodes are colored according to the cohort harboring the higher abundance for a definite species or cytokine, and node name size is directly proportional to the “keystonness” (importance of a species or cytokine within the overall network). Edge thickness is inversely proportional to the Pearson *p*-value after 10% Benjamini–Hochberg two-stages FDR, and it is colored according to positive (red) or negative (blue) Pearson coefficient. For each community are reported percentages of COVID-19- and controls-related nodes. Dashed line represents a “structural gap” (a bunch of negative Pearson correlation edges) between GREEN and RED communities. Correlogram **(B)** of bacterial species and cytokines shows positive (red) or negative (blue) Pearson correlation on normalized and standardized abundances. Significant correlation is marked with an asterisk inside each square: only species or cytokines having at least one significant correlation were reported. Dendrograms on the x and y axes were generated following Bray–Curtis similarity, evidencing three different clusters for bacterial species (shown here within white boxes). Cytokines chosen to compute the C4 parameter (IL-12p70, IL-6) are highlighted with a colored box in the top dendrogram. The parameter C4 computed for each species is depicted as bar plot length at the right of the correlogram, highlighting the bad species (in red) or the good ones (in green). Scatterplots **(C)** among the four selected species and the two cytokines used to compute the C4 parameter: their abundances are negatively related to one another (normalized and standardized data), as reported by Pearson coefficient and *p*-value (95% confidence interval, gray area). Kernel Density Estimation (KDE) plots **(D)** report on *X* axis the normalized and standardized abundance of the selected species and cytokines and on *Y* axis the subjects’ distribution, divided by COVID-19 (red) and controls (green).

Once we found that specific oral cytokines were distinctive for COVID-19, and that the oral cytokine profile was similar to the systemic circulation one, we made a cross-correlation and a network analysis aimed at finding functional clustering and relations among bacterial species and cytokines. Within the network, two distinct communities were formed, separated by a “structural gap” (bunch of negative Pearson correlations) (Figure 3A): the upper community (“GREEN”) harboring 86% of species or cytokines from controls and the lower one (“RED”) hosting 85% of species or cytokines having higher abundance in COVID-19 patients (χ^2^ = 20.5, *p* < 0.00001). Interestingly, keystone species in the GREEN community were *R. mucilaginosa* and *S. oralis*, already evidenced within the good community SIG3 (Figure 1D), while two cytokines (GMCSF and IL-4) were keystone within the RED community. Moreover, within RED cluster, we observed a sub-cluster of COVID-19-related species (*V. infantium*, *P. jejuni*, *Streptococcus cristatus*) already seen within the bad community SIG1 (Figure 1D) that were at the farthest distance from GREEN, thus highlighting their functional negative effect. In this proposition, given that unifying oral cytokine and species datasets crunched the overall network structure passing from four to two communities, and that a marked “structural gap” was evidenced among GREEN and RED communities (Figure 3A), the next step was to study all the possible correlations among the single species and the single cytokines, with the intention to highlight oral bacterial species with a potential to counteract COVID-19-related cytokines. An HCA correlogram based on Pearson correlation coefficients was performed, resulting in three different clusters of species based on their positive or negative correlation with cytokines (Figure 3B).

Cluster1 harbored bad species (such as *V. infantium*, *S. cristatus*, *Prevotella denticola*, and *A. parvulum*) already seen within COVID-19-related communities as SIG1, SIG4 (Figure 1D) and RED (Figure 3A). Cluster2 and Cluster3 contained mostly beneficial species that, conversely, were present within SIG2, SIG3 (Figure 1D) and GREEN (Figure 3A). With this notion in mind, and starting from Pearson coefficients (*r*) of the two cytokines distinctive for COVID-19 (IL-6) and controls (IL-12p70) (Figure 2D), we computed a parameter called C4 (COVID-19 Cytokines Counteracting Coefficient) valuable for each oral bacterial species (Figure 3B):

C4 = *r*_IL–12p70_ - *r*_IL–6_.

Averaging all C4 values within each cluster of the HCA correlogram resulted in C4_cluster1_ = −0.017, C4_cluster2_ = 0.472 and C4_cluster3_ = 0.301, with a significant difference among Cluster1 and Cluster2 (*t* = −2.72764, *p* = 0.0084, Figure 3B). Noteworthy, the species having the highest C4 values were *S. oralis*, *P. pasteri* and *R. mucilaginosa*, species previously found in SIG3 and GREEN communities (Supplementary Table 2). The detrimental species *V. infantium*, already found in the RED sub-cluster (Figure 3A) and within SIG1 (Figure 1D), had the highest negative C4 value, thus representing a plausible helper species for COVID-19 onset. In order to confirm the relation among these four species and the involved cytokines, we performed Pearson linear correlation scatterplots (Figure 3C) and Kernel Density Estimation (KDE) area plots (Figure 3D). Linear scatterplots confirmed the expected negative correlation among beneficial species and the pro-inflammatory IL-6, thus meaning that higher amounts of these bacteria could lower the pro-inflammatory oral environment. KDE plots measured patients’ distribution along the abundance of the four selected species or the two cytokines, evidencing how *R. mucilaginosa* and *P. pasteri*, having a clear superimposition of two peaks centered on the same value for COVID-19 (red) and controls (green), would act differently from *S. oralis*, which presents two green peaks mutually excludable from the single red one (Figure 3D). The information provided by KDE plots would thus be compulsory for a plausible fine-tuning regulation of species relative abundances in the oral cavity against COVID-19 onset. Taking into consideration the results from Figures 1, 3, we selected three species as potential counteractors of COVID-19, namely, *S. oralis*, *R. mucilaginosa* and *P. pasteri*. These species topologically grouped within SIG3, together with other possible candidates considerable as “helpers” to their positive function: *Granulicatella adiacens*, *G. elegans*, *G. taiwanensis*, and *N. perflava*. The collective information gathered from network analysis, HCA correlograms and KDE plots integrating species and cytokines datasets would thus be amenable for a specific probiotic formulation committed against COVID-19 onset and/or COVID-19-related cytokines.

### Consortia of Bacteria and Cytokines Predict COVID-19 Status and Ageusia/Hyposmia

**FIGURE 4:**
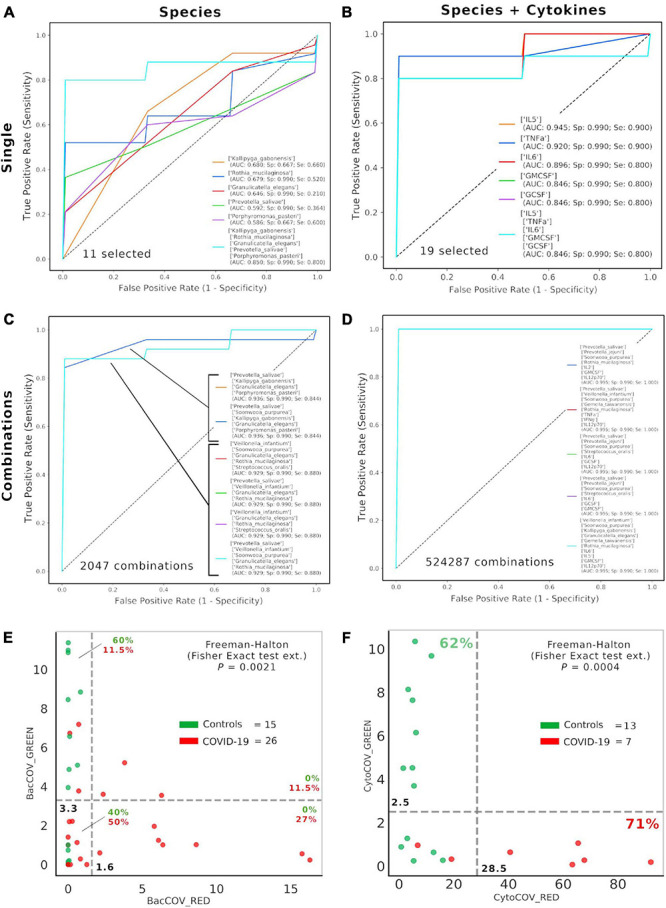
Consortia of bacteria and cytokines predict COVID-19 status. Receiver Operating Characteristic (ROC) with fivefold cross-validation and C-Support Vector Machine classifier was employed to assess the best oral bacterial species (among 11 selected, **(A)** or species plus cytokines (among 19 selected, **(B)** able to predict COVID-19 status. Each panel reports the best five Area Under Curve (AUC) values in descending order (see the inset legend also showing specificity, Sp, and sensitivity, Se, for each ROC curve), plus a sixth curve encompassing the preceding five grouped. Combinations were computed for selected species (*n* = 11, 2,047 combinations, **(C)** and species plus cytokines (*n* = 19, 524,287 combinations, **(D)**, and the best “consortia” predicting COVID-19 are shown along with their AUC, Sp and Se values. BacCOV **(E)** and CytoCOV **(F)** parameters were computed and divided into their GREEN (species and cytokines higher in controls) and RED (species and cytokines higher in COVID-19) components and employed to generate scatterplot 2D graphs. Abundance thresholds (computed as in Supplementary Table 5) are shown as dotted gray lines, and their values are reported in bold. In each quadrant of panels E and F are reported the percentages of controls (green) or COVID-19 subjects (red).

After selecting 11 bacterial species (Figure 1) and 8 cytokines (Figure 2) as biomarkers for COVID-19 and controls, along with their topological relationships (Figure 3), we focused our attention to their predictive power. Employing the Receiver Operating Characteristic (ROC) metric to evaluate classifier output quality using a fivefold cross-validation, we evidenced how single cytokines gave a higher power when unified to single species in predicting COVID-19 status (Figures 4A,B), showing a significant higher averaged Area Under Curve (AUC) (AVG_AUCavg(Cytokines_Species)_ = 0.891; AVG_AUCavg(Species)_ = 0.637; Mann–Whitney *U* test, two-tailed *p* = 0.012). Moreover, the first five ROC curves in Figure 2B, representing the ROCs having the best AUC values, were devoted to only cytokines, thus confirming their importance in defining the disease status better than species. Aiming at finding the best consortium of bacterial species (*n* = 11) or species and cytokines (*n* = 19) able to predict the COVID-19 status, a combinatorial calculation was performed (Supplementary Table 3). A total of 2,047 and 524,287 combinations were retrieved for species (Figure 4C) and species plus cytokines (Figure 4D), respectively, and also in this scenario, the best averaged AUC value was significantly higher (Mann–Whitney *U* test, two-tailed *p* = 0.012) for “consortia” of species plus cytokines (AVG_AUCavg(Cytokines_Species)_ = 0.995), other than considering proper consortia formed by species alone (AVG_AUCavg(Cytokines_Species)_ = 0.932). Interestingly, the majority of consortia showing ROC curves with the highest values of AUC, specificity and sensitivity (namely, 0.995, 0.990, and 1.000, respectively, Figure 4D) were those encompassing a balanced ratio of species and cytokines that were beneficial (*P. salivae*, *S. oralis*, *R. mucilaginosa*, *G. taiwanensis*, *K. gabonensis*, *G. elegans*, IL-12p70) or detrimental (*P. jejuni*, *S. purpurea*, *V. infantium*, TNF-α, INF-γ, IL-2, IL-6, IL-5, GCSF, and GMCSF). After assessing that definite combinations of our selected bacterial species, alone or added to oral cytokines, were able to discriminate COVID-19 status, we aimed at parametrizing each single subject for a general COVID-19 susceptibility. To this aim, we created two parameters, named BacCOV and CytoCOV, based on the abundances of selected bacterial species or cytokines (shown in Figures 1G, 2D). More precisely, in order to have a bidimensional representation for both cohorts, usable to visually predict the propensity of a putative unknown subject to COVID-19, we computed for each subject: (i) the BacCOV_GREEN and CytoCOV_GREEN values averaging the abundances of beneficial species (*n* = 7) or species plus cytokines (*n* = 7+1) and (ii) the BacCOV_RED and CytoCOV_RED values averaging the abundances of detrimental species (*n* = 4) or species plus cytokines (*n* = 4+7) (Supplementary Table 4). These four values were used to generate L-shaped graphs, in which we set *X*-axis and *Y*-axis thresholds (computed as in Supplementary Table 5) in order to ease a clinical usage for subjects’ COVID-19 susceptibility (Figures 4E,F). Even if BacCOV significantly divided the two cohorts (*p* = 2.1^∗^10^–3^), a one-order higher significant separation was obtained with CytoCOV (*p* = 4^∗^10^–4^), correctly classifying 71% of COVID-19 patients (Figure 4F). Thus, the CytoCOV parameter would be easily employed in clinics to assess COVID-19 susceptibility for an unknown subject, through the following passages: (i) assaying the abundances of 11 bacterial species and 8 cytokines; (ii) computing the CytoCOV parameter (Supplementary Table 4); and (iii) using the boundaries provided in Table 2.

**TABLE 2:** Boundaries for CytoCOV parameter, as evidenced in Figure 4F, in order to assess subjects’ COVID-19 predicted susceptibility.

	**CytoCOV_RED ≥ 28.5 CytoCOV_GREEN < 2.5**	**CytoCOV_RED < 28.5 CytoCOV_GREEN ≥ 2.5**	**CytoCOV_RED < 28.5 CytoCOV_GREEN < 2.5**
Controls	0%	62%	38%
COVID-19	71%	0%	29%

*Percentage values represent subjects’ fraction.*

**FIGURE 5:**
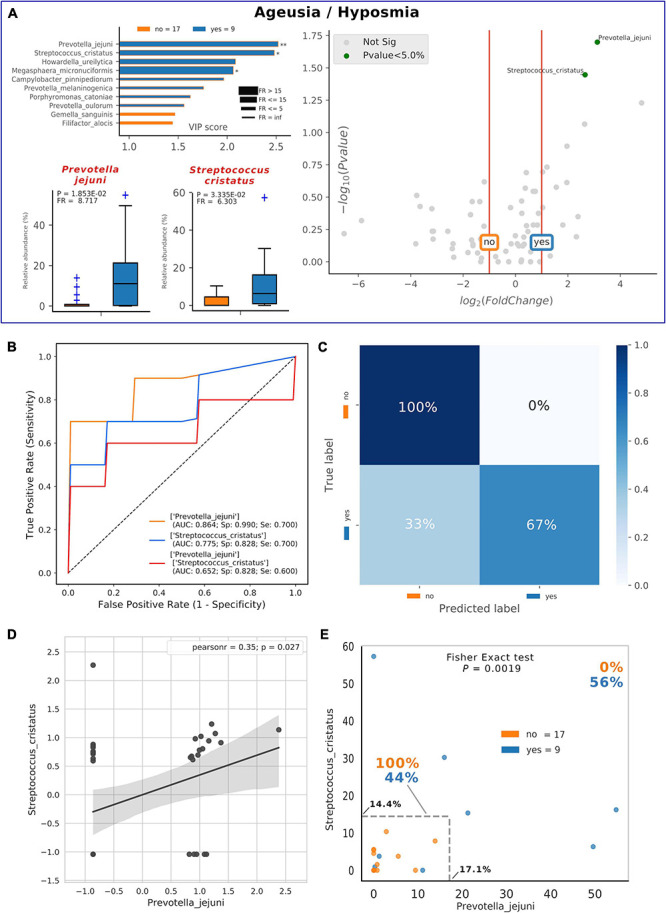
Consortia of bacteria predict COVID-19 ageusia/hyposmia. Mixed graphs for COVID-19 patients having ageusia/hyposmia **(A)** are made of VIP plot, volcano plot and boxplot of relative abundances for selected species. Description for these plots as in Figures 1, 2. Significant difference after Mann–Whitney U test (non-FDR, *p < = 0.05, **p < = 0.01). Receiver Operating Characteristic (ROC) with fivefold cross-validation and C-Support Vector Machine classifier **(B)** was built for each one of the selected species plus their combined effect on predicting the presence of ageusia/hyposmia (“yes,” blue) in respect to their absence (“no,” orange). Panel B reports Area Under Curve (AUC), specificity (Sp), and sensitivity (Se) values for each ROC curve. Confusion matrix **(C)** was employed to evaluate the quality of the output of the C-Support Vector Machine classifier used to generate ROC curves. The diagonal elements represent the percentage of patients (referred to the blue shadowed sidebar) for which the predicted label (*X* axis) is equal to the true label (*Y* axis), while off-diagonal elements are those that are mislabeled by the classifier. Scatterplot **(D)** among the two selected species shows how their relative abundances are positively related to one another (normalized and standardized data), as reported by Pearson coefficient and *p*-value (95% confidence interval, gray area). Raw relative abundances of selected species were used to generate a scatterplot **(E)** to ease a clinical interpretation of patients’ distribution into the 2D space. Abundance thresholds are shown as truncated dotted gray lines, and their values are reported in bold: percentages of patients separated by these boundaries are reported in bold.

Thirty-five percent of our COVID-19 patients (9/26) presented, as comorbidity, ageusia and/or hyposmia. Applying the same analysis used previously, even if these patients did not possess a distinctive oral microbiota composition (data not shown), significant higher levels of detrimental species such as *P. jejuni* and *S. cristatus* were found (Figure 5A). Interestingly, these species were found within the RED community in the combined species/cytokines network (Figure 3A). Averaged AUC values of ROC curves regarding these two selected species were 0.864 and 0.775, respectively (Figure 5B), while their combination ensured an accurate prediction of 67% of patients with ageusia/hyposmia (confusion matrix, Figure 5C). Interestingly, these two species were positively and significantly related (*r* = 0.35, *p* = 0.027, Figure 5D), so we employed a bidimensional representation of their relative abundances in order to assess patients’ ageusia/hyposmia susceptibility (Figure 5E). An unknown COVID-19 patient would thus be susceptible of ageusia/hyposmia if harboring *P. jejuni* and *S. cristatus* at relative abundances higher than or equal to 17.1% or 14.4%, respectively (Fisher’s exact test *p* = 1.9^∗^10^–3^).

## Discussion

COVID-19 proved to be an important threat to our lives, harnessing more than 60 million cases worldwide and around 1.5 million global deaths, as of November 2020 (Dong et al., 2020). The major issue when coping with the COVID-19 etiological agent, SARS-CoV-2 betacoronavirus, resides in its high spreading ability, which is mediated by several interpersonal factors, such as oral droplets (Bao et al., 2020; Netz and Eaton, 2020). In this proposition, the oral cavity should be considered a preferential route for SARS-CoV-2 entry or transmission (Jiao et al., 2020; Netz and Eaton, 2020), especially in nosocomial environments at risk (Kumbargere Nagraj et al., 2020), with potential involvement for extrapulmonary sites (such as the brain (Gupta et al., 2020; Kanberg et al., 2020) and gastrointestinal tract) (Jiao et al., 2020; Trottein and Sokol, 2020). Here, we investigated the oral microbiota composition and cytokines in severe COVID-19 subjects and matched controls. As of January 2020, only a few experimental studies focused on the COVID-19-related intestinal bacterial microbiota (Gu et al., 2020; Zuo et al., 2020a, b) and on fungal one (Zuo et al., 2020c), leaving unanswered questions on the oral district. The new approach of social network analysis allowed us to properly merge species and cytokines datasets, providing definite bacterial consortia as biomarkers for COVID-19 status or ageusia/hyposmia and providing also new parameters for clinical purposes against COVID-19. Our results especially suggest how a minimal consortium of beneficial species (*P. salivae*, *S. oralis*, *R. mucilaginosa*, *G. taiwanensis*, *K. gabonensis*, *G. elegans*) could be used orally as local probiotics to counteract COVID-19 symptoms and cytokine storm, which is typical in severe COVID-19 patients (Guo et al., 2020; Jose and Manuel, 2020). In a recent study, genera *Rothia*, *Streptococcus* and *Veillonella* were positively related to COVID-19 in feces (Gu et al., 2020), while here, we found how some oral species belonging to these genera exert a counteracting effect on COVID-19 cytokine storm (Jose and Manuel, 2020). This discrepancy is noteworthy because future studies dealing with different body districts should consider shotgun sequencing or 16S-targeted sequencing to reach the species level, assuring a proper functional description (e.g., combination of microbiota and cytokine data) for clinical use (Manna et al., 2020; Peddu et al., 2020). Being attractive for the ease of sampling, as demonstrated in international projects such as HMP, the oral swab sampling (touching tongue, palatum and cheeks) would be a valid alternative to the currently used specimens (e.g., feces and blood) to assess the microbiota compositional differences in a disease, giving reliable insights on a subject susceptibility. Our study has intrinsic limitations: (i) the low number of subjects involved; (ii) the missing point of a shotgun implementation to ascertain gene and/or pathways; and (iii) we could not be sure if the observed dysbiosis was already in action in COVID-19 patients when oral swab samples were taken. Based on the results of this pilot study, we are planning a forthcoming research encompassing more patients to be recruited, trying to correlate symptoms onset and SARS-CoV-2 viral loads in a time-course fashion. In the forthcoming workflow, there is a thorough description of the local microbiota and its functional inference through metagenomics [as performed in our previous study on oral Neisseriaceae (Donati et al., 2016)], along with direct isolation and whole-genome sequencing (WGS) of resident species important for the local homeostatic ecology (such as *Neisseria* spp.) or of species already established in pre-existing pathological conditions (such as *Prevotella* spp. and *Veillonella* spp.) (Leishman et al., 2010; Mammen et al., 2020). Even with these limitations, our study would give a hint to the importance of oral microbiota modulation for COVID-19 symptoms treatment or detection (Al-Khatib, 2020; Bao et al., 2020; Chen et al., 2020b; Patel and Sampson, 2020; Sampson et al., 2020).

## Data Availability Statement

The datasets presented in this study can be found in online repositories. The names of the repository/repositories and accession number(s) can be found below: https://www.ncbi.nlm.nih.gov/, PRJNA692359.

## Ethics Statement

The studies involving human participants were reviewed and approved by University of Trieste Ethical Committee. The patients/participants provided their written informed consent to participate in this study.

## Author Contributions

VI conceived the article, performed the bioinformatic and statistical analysis, and wrote the manuscript. NZ, GC, and CC performed the experimental analysis on sequencing and cytokines quantification. VZ, SD, RL, MC, and AP provided clinical samples and patients’ metadata. MC conceived the article and wrote the article. All authors contributed to the manuscript optimization.

## Conflict of Interest

The authors declare that the research was conducted in the absence of any commercial or financial relationships that could be construed as a potential conflict of interest.

## Publisher’s Note

All claims expressed in this article are solely those of the authors and do not necessarily represent those of their affiliated organizations, or those of the publisher, the editors and the reviewers. Any product that may be evaluated in this article, or claim that may be made by its manufacturer, is not guaranteed or endorsed by the publisher.

## References

[B1] Al-Khatib A. (2020). Oral manifestations in COVID-19 patients. *Oral Dis.* 27 Suppl 3 779–780. 10.1111/odi.13477 32521067 PMC7307069

[B2] Anderson M. J. Walsh D. C. I. (2013). PERMANOVA, ANOSIM, and the Mantel test in the face of heterogeneous dispersions: What null hypothesis are you testing? *Ecol. Monogr.* 83 557–574. 10.1890/12-2010.1

[B3] Bao L. Zhang C. Dong J. Zhao L. Li Y. Sun J. (2020). Oral Microbiome and SARS-CoV-2: beware of lung co-infection. *Front. Microbiol.* 11:1840. 10.3389/fmicb.2020.01840 32849438 PMC7411080

[B4] Berry D. Widder S. (2014). Deciphering microbial interactions and detecting keystone species with co-occurrence networks. *Front. Microbiol.* 5:219. 10.3389/fmicb.2014.00219 24904535 PMC4033041

[B5] Blondel V. D. Guillaume J.-L. Lambiotte R. Lefebvre E. (2008). Fast unfolding of communities in large networks. *J. Stat. Mech. Theor. Exp.* 2008:10008. 10.1088/1742-5468/2008/10/p10008

[B6] Braz-Silva P. H. Pallos D. Giannecchini S. To K. K. (2020). SARS-CoV-2: what can saliva tell us? *Oral Dis.* 27 Suppl 3 746–747. 10.1111/odi.13365 32311181 PMC7264628

[B7] Campisciano G. Toschetti A. Comar M. Taranto R. D. Berton F. Stacchi C. (2017). Shifts of subgingival bacterial population after nonsurgical and pharmacological therapy of localized aggressive periodontitis, followed for 1 year by Ion Torrent PGM platform. *Eur. J. Dent.* 11 126–129. 10.4103/ejd.ejd_309_16 28435379 PMC5379826

[B8] Chen X. Liao B. Cheng L. Peng X. Xu X. Li Y. (2020a). The microbial coinfection in COVID-19. *Appl. Microbiol. Biotechnol.* 104 7777–7785. 32780290 10.1007/s00253-020-10814-6 PMC7417782

[B9] Chen X. Zhao B. Qu Y. Chen Y. Xiong J. Feng Y. (2020b). Detectable serum SARS-CoV-2 viral load (RNAaemia) is closely associated with drastically elevated interleukin 6 (IL-6) level in critically ill COVID-19 patients. *medRxiv* [Preprint] 10.1101/2020.02.29.20029520 PMC7184354 32301997

[B10] de la Rica R. Borges M. Gonzalez-Freire M. (2020). COVID-19: in the eye of the cytokine storm. *Front. Immunol* 11:558898. 10.3389/fimmu.2020.558898 33072097 PMC7541915

[B11] Derosa L. Routy B. Fidelle M. Iebba V. Alla L. Pasolli E. (2020). Gut bacteria composition drives primary resistance to cancer immunotherapy in renal cell carcinoma patients. *Eur. Urol.* 78 195–206. 10.1016/j.eururo.2020.04.044 32376136

[B12] Donati C. Zolfo M. Albanese D. Tin Truong D. Asnicar F. Iebba V. (2016). Uncovering oral Neisseria tropism and persistence using metagenomic sequencing. *Nat. Microbiol.* 1:16070. 10.1038/nmicrobiol.2016.70 27572971

[B13] Dong E. Du H. Gardner L. (2020). An interactive web-based dashboard to track COVID-19 in real time. *Lancet Infect. Dis.* 20 533–534. 10.1016/s1473-3099(20)30120-1 32087114 PMC7159018

[B14] Faust K. Raes J. (2012). Microbial interactions: from networks to models. *Nat. Rev. Microbiol.* 10 538–550. 10.1038/nrmicro2832 22796884

[B15] Faust K. Sathirapongsasuti J. F. Izard J. Segata N. Gevers D. Raes J. (2012). Microbial co-occurrence relationships in the human microbiome. *PLoS Comput. Biol.* 8:e1002606. 10.1371/journal.pcbi.1002606 22807668 PMC3395616

[B16] Feng Z. Wang Y. Qi W. (2020). The small intestine, an underestimated site of SARS-CoV-2 infection: from red queen effect to probiotics. *Preprints* 10.20944/preprints202003.0161.v1 32283112

[B17] Gu S. Chen Y. Wu Z. Chen Y. Gao H. Lv L. (2020). Alterations of the gut microbiota in patients with coronavirus disease 2019 or H1N1 influenza. *Clin. Infect. Dis.* 71 2669–2678. 10.1093/cid/ciaa709 32497191 PMC7314193

[B18] Guo Y.-R. Cao Q.-D. Hong Z.-S. Tan Y.-Y. Chen S.-D. Jin H.-J. (2020). The origin, transmission and clinical therapies on coronavirus disease 2019 (COVID-19) outbreak - an update on the status. *Mil. Med. Res.* 7:11. 10.1186/s40779-020-00240-0 PMC7068984 32169119

[B19] Gupta A. Madhavan M. V. Sehgal K. Nair N. Mahajan S. Sehrawat T. S. (2020). Extrapulmonary manifestations of COVID-19. *Nat. Med.* 26 1017–1032. 32651579 10.1038/s41591-020-0968-3 PMC11972613

[B20] Herrera D. Serrano J. Roldán S. Sanz M. (2020). Is the oral cavity relevant in SARS-CoV-2 pandemic? *Clin. Oral Investig.* 24 2925–2930. 10.1007/s00784-020-03413-2 32577830 PMC7309196

[B21] Iebba V. Guerrieri F. Di Gregorio V. Levrero M. Gagliardi A. Santangelo F. (2018). Combining amplicon sequencing and metabolomics in cirrhotic patients highlights distinctive microbiota features involved in bacterial translocation, systemic inflammation and hepatic encephalopathy. *Sci. Rep.* 8:8210. 10.1038/s41598-018-26509-y PMC5974022 29844325

[B22] Jiao L. Li H. Xu J. Yang M. Ma C. Li J. (2020). The gastrointestinal tract is an alternative route for SARS-CoV-2 infection in a nonhuman primate model. *Gastroenterology* 160 1647–1661. 10.1053/j.gastro.2020.12.001 33307034 PMC7725054

[B23] Jose R. J. Manuel A. (2020). COVID-19 cytokine storm: the interplay between inflammation and coagulation. *Lancet Respir. Med.* 8 e46–e47. 32353251 10.1016/S2213-2600(20)30216-2 PMC7185942

[B24] Kanberg N. Ashton N. J. Andersson L.-M. Yilmaz A. Lindh M. Nilsson S. (2020). Neurochemical evidence of astrocytic and neuronal injury commonly found in COVID-19. *Neurology* 95 e1754–e1759. 32546655 10.1212/WNL.0000000000010111

[B25] Kumbargere Nagraj S. Eachempati P. Paisi M. Nasser M. Sivaramakrishnan G. Verbeek J. H. (2020). Interventions to reduce contaminated aerosols produced during dental procedures for preventing infectious diseases. *Cochrane Database Syst. Rev.* 10:CD013686. 10.1002/14651858.CD013686.pub2 PMC8164845 33047816

[B26] Lambiotte R. Delvenne J.-C. Barahona M. (2014). Random walks, markov processes and the multiscale modular organization of complex networks. *IEEE Trans. Network Sci. Eng.* 1 76–90. 10.1109/tnse.2015.2391998

[B27] Leishman S. J. Do H. L. Ford P. J. (2010). Cardiovascular disease and the role of oral bacteria. *J. Oral Microbiol.* 2 10.3402/jom.v2i0.5781 21523220 PMC3084572

[B28] Leung E. C. Chow V. C. Lee M. K. Lai R. W. (2020). Deep throat saliva as an alternative diagnostic specimen type for the detection of SARS-CoV-2. *J. Med. Virol.* 93 533–536. 10.1002/jmv.26258 32621616 PMC7361401

[B29] Li J. Gong X. Wang Z. Chen R. Li T. Zeng D. (2020). Clinical features of familial clustering in patients infected with 2019 novel coronavirus in Wuhan, China. *Virus Res.* 286:198043. 10.1016/j.virusres.2020.198043 32502551 PMC7265838

[B30] Li M. Wang B. Zhang M. Rantalainen M. Wang S. Zhou H. (2008). Symbiotic gut microbes modulate human metabolic phenotypes. *Proc. Natl. Acad. Sci. U.S.A.* 105 2117–2122. 18252821 10.1073/pnas.0712038105 PMC2538887

[B31] Lozupone C. A. Stombaugh J. I. Gordon J. I. Jansson J. K. Knight R. (2012). Diversity, stability and resilience of the human gut microbiota. *Nature* 489 220–230. 10.1038/nature11550 22972295 PMC3577372

[B32] Ma D. Chen C.-B. Jhanji V. Xu C. Yuan X.-L. Liang J.-J. (2020). Expression of SARS-CoV-2 receptor ACE2 and TMPRSS2 in human primary conjunctival and pterygium cell lines and in mouse cornea. *Eye* 34 1212–1219. 10.1038/s41433-020-0939-4 32382146 PMC7205026

[B33] Mammen M. J. Scannapieco F. A. Sethi S. (2020). Oral-lung microbiome interactions in lung diseases. *Periodontology 2000* 83 234–241. 10.1111/prd.12301 32385873

[B34] Manna S. Baindara P. Mandal S. M. (2020). Molecular pathogenesis of secondary bacterial infection associated to viral infections including SARS-CoV-2. *J. Infect. Public Health* 13 1397–1404. 10.1016/j.jiph.2020.07.003 32712106 PMC7359806

[B35] Merico D. Gfeller D. Bader G. D. (2009). How to visually interpret biological data using networks. *Nat. Biotechnol.* 27 921–924. 10.1038/nbt.1567 19816451 PMC4154490

[B36] Netz R. R. Eaton W. A. (2020). Physics of virus transmission by speaking droplets. *Proc. Natl. Acad. Sci. U.S.A.* 117 25209–25211. 10.1073/pnas.2011889117 32973098 PMC7568337

[B37] Patel J. Sampson V. (2020). The role of oral bacteria in COVID-19. *Lancet Microbe* 1:e105. 10.1016/s2666-5247(20)30057-4 PMC7333982 32835339

[B38] Peddu V. Shean R. C. Xie H. Shrestha L. Perchetti G. A. Minot S. S. (2020). Metagenomic analysis reveals clinical SARS-CoV-2 infection and bacterial or viral superinfection and colonization. *Clin. Chem.* 66 966–972. 10.1093/clinchem/hvaa106 32379863 PMC7239240

[B39] Qin C. Zhou L. Hu Z. Zhang S. Yang S. Tao Y. (2020). Dysregulation of immune response in patients with COVID-19 in Wuhan, China. *SSRN Electron. J.* 10.2139/ssrn.3541136 PMC7108125 32161940

[B40] Raghu G. Wilson K. C. (2020). COVID-19 interstitial pneumonia: monitoring the clinical course in survivors. *Lancet Respir. Med.* 8 839–842. 10.1016/s2213-2600(20)30349-0 32758440 PMC7398671

[B41] Riganelli L. Iebba V. Piccioni M. Illuminati I. Bonfiglio G. Neroni B. (2020). Structural variations of vaginal and endometrial microbiota: hints on female infertility. *Front. Cell. Infect. Microbiol.* 10:350. 10.3389/fcimb.2020.00350 32760681 PMC7372811

[B42] Sampson V. Kamona N. Sampson A. (2020). Could there be a link between oral hygiene and the severity of SARS-CoV-2 infections? *Br. Dent. J.* 228 971–975. 10.1038/s41415-020-1747-8 32591714 PMC7319209

[B43] Srivastava P. Gupta N. (2020). Clinical manifestations of corona virus disease. *Clin. Syn. COVID-19* 31–49. 10.1007/978-981-15-8681-1_3

[B44] To K. K.-W. Tsang O. T.-Y. Leung W.-S. Tam A. R. Wu T.-C. Lung D. C. (2020a). Temporal profiles of viral load in posterior oropharyngeal saliva samples and serum antibody responses during infection by SARS-CoV-2: an observational cohort study. *Lancet Infect. Dis.* 20 565–574. 10.1016/s1473-3099(20)30196-1 32213337 PMC7158907

[B45] To K. K.-W. Tsang O. T.-Y. Yip C. C.-Y. Chan K.-H. Wu T.-C. Chan J. M.-C. (2020b). Consistent detection of 2019 novel coronavirus in saliva. *Clin. Infect. Dis.* 71 841–843. 32047895 10.1093/cid/ciaa149 PMC7108139

[B46] Trottein F. Sokol H. (2020). Potential causes and consequences of gastrointestinal disorders during a SARS-CoV-2 infection. *Cell Rep.* 32:107915. 10.1016/j.celrep.2020.107915 32649864 PMC7332457

[B47] Wang D. Hu B. Hu C. Zhu F. Liu X. Zhang J. (2020). Clinical characteristics of 138 hospitalized patients with 2019 novel coronavirus-infected pneumonia in Wuhan, China. *JAMA* 323 1061–1069. 10.1001/jama.2020.1585 32031570 PMC7042881

[B48] Wong A. W. Fidler L. Marcoux V. Johannson K. A. Assayag D. Fisher J. H. (2020). Practical considerations for the diagnosis and treatment of fibrotic interstitial lung disease during the coronavirus disease 2019 pandemic. *Chest* 158 1069–1078. 10.1016/j.chest.2020.04.019 32333929 PMC7194738

[B49] Xiang Z. Koo H. Chen Q. Zhou X. Liu Y. Simon-Soro A. (2020). Potential implications of SARS-CoV-2 oral infection in the host microbiota. *J. Oral Microbiol.* 13:1853451. 10.1080/20002297.2020.1853451 33312449 PMC7711743

[B50] Zanotta N. Maximova N. Campisciano G. Del Savio R. Pizzol A. Casalicchio G. (2015). Up-regulation of the monocyte chemotactic protein-3 in sera from bone marrow transplanted children with torquetenovirus infection. *J. Clin. Virol.* 63 6–11. 10.1016/j.jcv.2014.11.028 25600596

[B51] Zuo T. Liu Q. Zhang F. Lui G. C.-Y. Tso E. Y. Yeoh Y. K. (2020a). Depicting SARS-CoV-2 faecal viral activity in association with gut microbiota composition in patients with COVID-19. *Gut* 70 276–284. 10.1136/gutjnl-2020-322294 32690600 PMC7385744

[B52] Zuo T. Zhan H. Zhang F. Liu Q. Tso E. Y. K. Lui G. C. Y. (2020c). Alterations in fecal fungal microbiome of patients with COVID-19 during time of hospitalization until discharge. *Gastroenterology* 159 1302–1310.e5. 10.1053/j.gastro.2020.06.048 32598884 PMC7318920

[B53] Zuo T. Zhang F. Lui G. C. Y. Yeoh Y. K. Li A. Y. L. Zhan H. (2020b). Alterations in gut microbiota of patients with COVID-19 during time of hospitalization. *Gastroenterology* 159 944–955.e8. 10.1053/j.gastro.2020.05.048 32442562 PMC7237927

