# Supplementary figures and images for: Profiling of Oral Microbiota and Cytokines in COVID-19 Patients
Source: Front Microbiol. 2021 Jul 30;12:671813. doi: 10.3389/fmicb.2021.671813 (PMC8361794; doi:10.3389/fmicb.2021.671813)

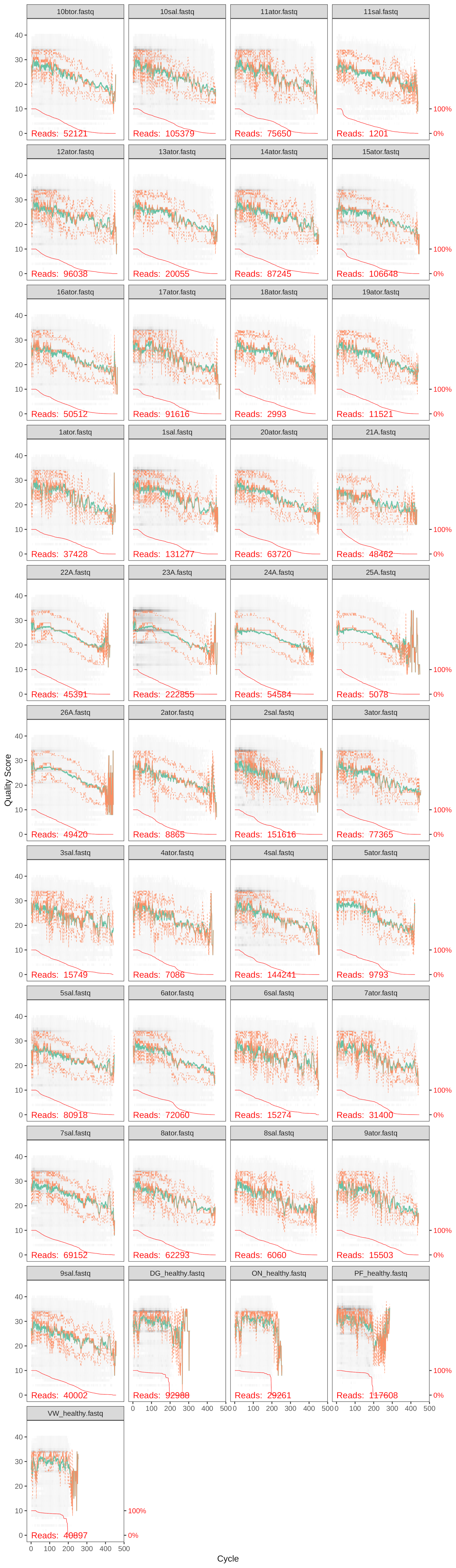

Supplement: Supplementary file 6 [file Image_1.TIF]

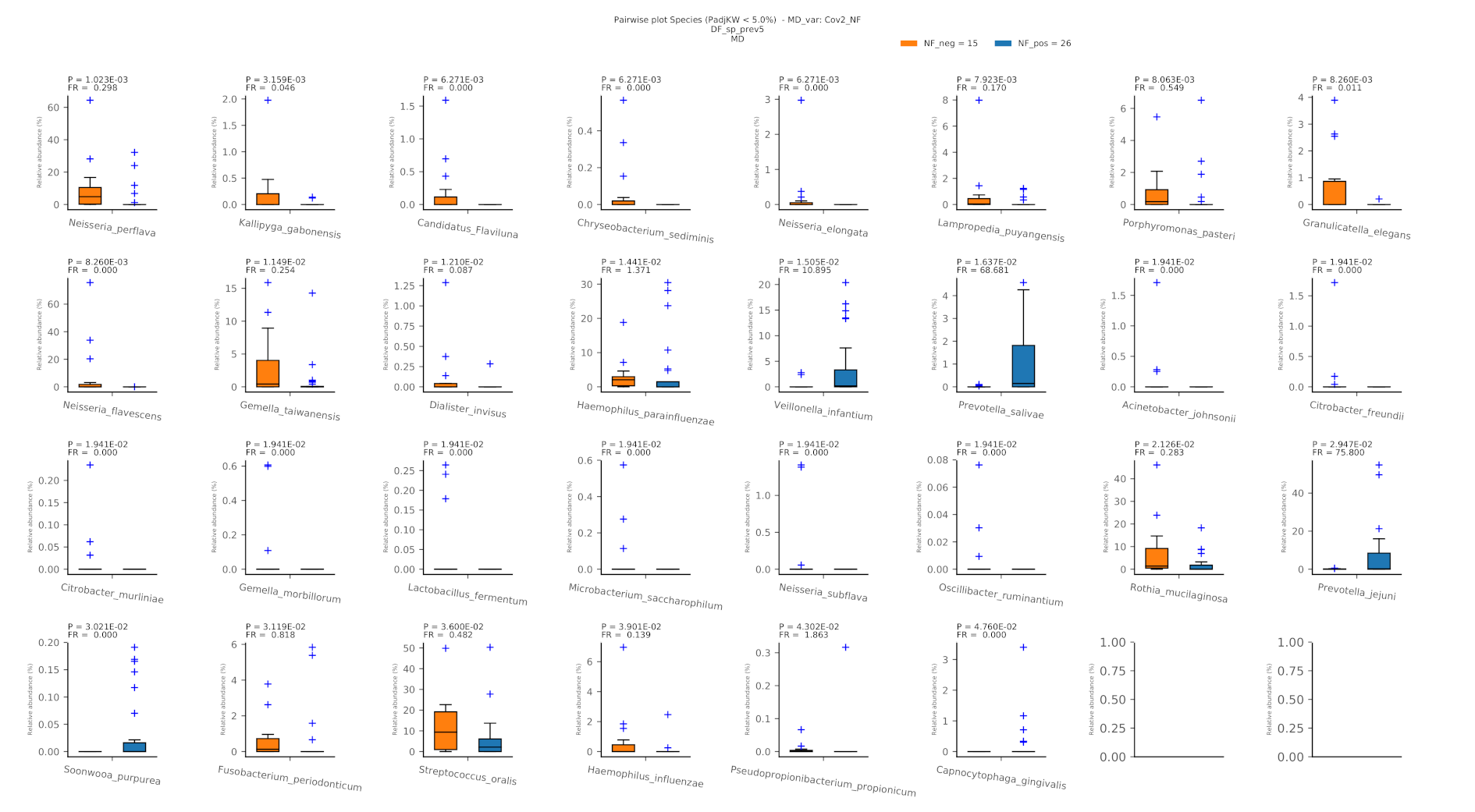

Supplement: Supplementary file 7 [file Image_2.TIF]

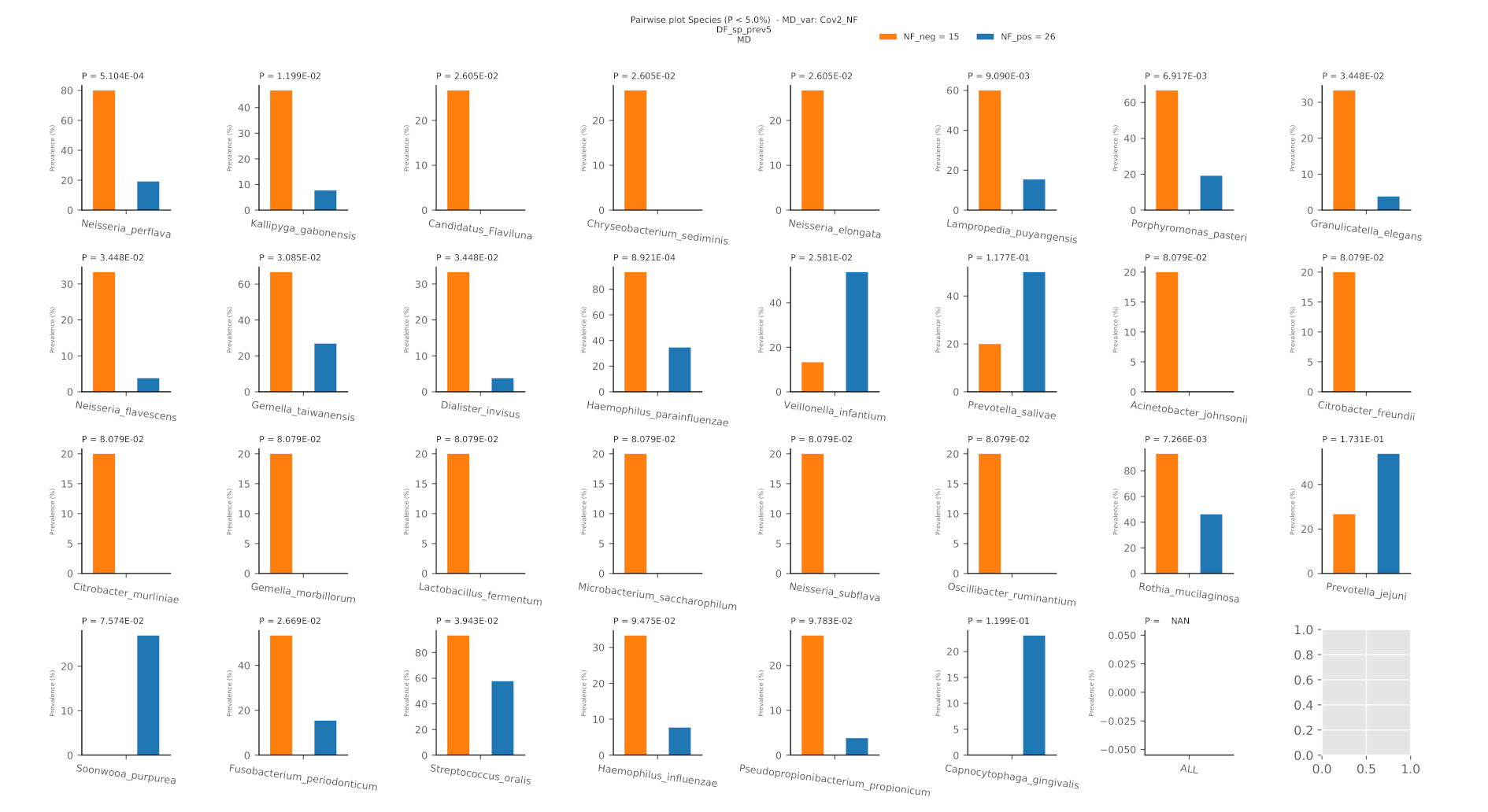

Supplement: Supplementary file 8 [file Image_3.TIF]

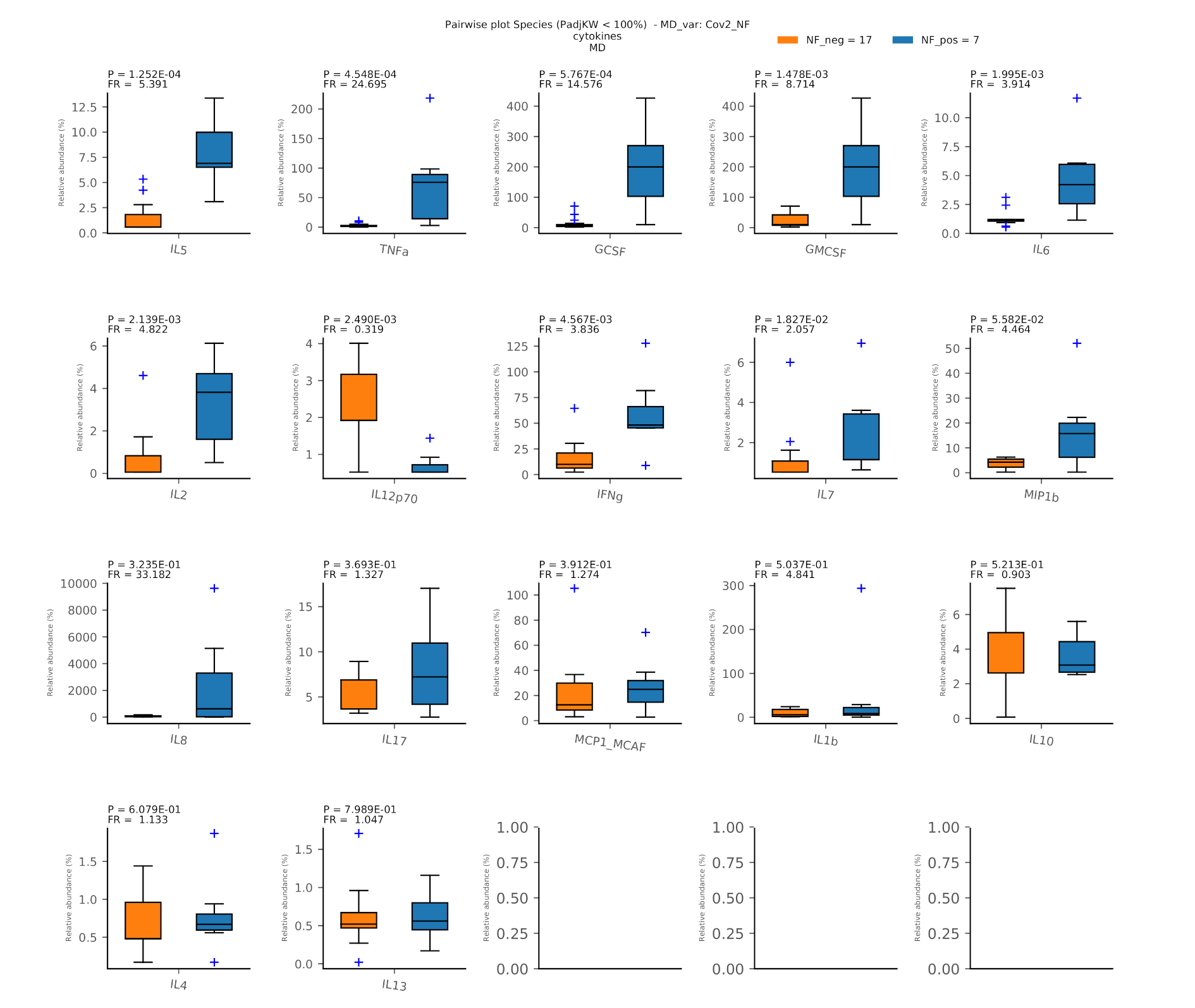

Supplement: Supplementary file 9 [file Image_4.TIF]
